# To the Editors of the Medical and Physical Journal

**Published:** 1807-10

**Authors:** G. Bellamy

**Affiliations:** His Majesty's Ship Hector, Plymouth


### *To the Editors of the Medical and Physical Journal.*

GENTLEMEN,

WILL you do me the favour of inserting the following Case of Calculus in the Urethra, and that of a Lusus Naturæ; an instance of the kind, to the best of my knowledge, is not recorded; and it is allowed to be essential in all the studies of Nature to be aware of her aberrations.

I am, &c.

G. BELLAMY, M. D.

*His Majesty's Ship Hector, Plymouth, August 24th*, 1807.

---

Henri Lotier, a French prisoner of war, came under my care, October 16, 1806, for a calculus in the urethra; very large, lodged about half an inch from the orifice of the glans penis. I tried in vain with various instruments to extract it; then divided the urethra in a line with the orifice, in preference to the part immediately over the stone, being less liable to become fistulous or callous, and more easily brought in contact, and only one passage for the urine. The stone weighed 9 grs.; I succeeded in healing the wound more readily than I expected, and with less inflammation; by the help of the bougie, adhesive straps, saturne lotions, occasional cathartics, and opiates; but the orifice remained about half more than its natural size, and fortunately that it was so, and that I preferred that part to make it; for about six months after, he evacuated, after a little suffering, one much larger, weighing gr. xiii ¼. Now, as there is a predisposition to calculi in such patients, I should in future cases divide at the same spot, provided the calculus could be brought forward, even within an inch of the orifice, and I would intentionally leave it rather larger than natural. The advantages of which plan are too plain to be enumerated, as are also incoveniences attendant on repeated incision of the urethra, for each stone which might be presented.

Jean Baptiste Fournier, aged about 50 years, has been from his birth in a state of entire emasculation; he first presented himself to me an invalid, with a whisper, "Monsieur je ne suis pas homme." I observed a degree of effeminacy in his manner, figure, and complexion; but his voice less so than the other marks. Skin fair, thin, and soft; muscles of a lax fibre, and the chin beardless. He

He was embarked as a sailor, but had little nerve or masculine vigour of mind or body. On exhibiting the pubes the appearances were perfectly infantile, of the male kind; penis exceedingly small, with a long prepuce, in all about an inch and a half, but perfect in figure and parts, if an infant; with some difficulty, a part of the glans may be exposed, and the orifice of the urethra is natural. The scrotum is very small, even less than that of a fœtus, and is flattened on the pubes, and appears with the penis, to be situated much higher on the pubes than usual. There is not one hair, nor, by the most minute examination, the least trace of testes, or spermatic vessels; he has occasionally slight erections, but never any seminal evacuation, or uneasiness for want of venereal enjoyment, the desire for which he never felt; in short, his own expression, "Jai passé une triste jeunesse" is the best comment on his degree of manhood.

